# Supplementary material for: What is an archaeon and are the Archaea really unique?
Source: PeerJ. 2018 Oct 18;6:e5770. doi: 10.7717/peerj.5770 (PMC6196074; doi:10.7717/peerj.5770)
Supplement: Supplemental Information 1 [file peerj-06-5770-s001.pdf]

Supplementary Material for

What is an Archaeon and are the Archaea Really Unique?

Ajith Harish

Department of Cell and Molecular Biology, Section of Structural and Molecular Biology, Uppsala University, Uppsala, Sweden

Table of Contents……………………………………………………………….. page #

Supplementary Methods…………………………………………………………….. 02
Supplementary References………………………………………………………..… 05
Supplementary Figures...…………………………………………………………..… 06
Supplementary Tables………………………………………………………………… 08

SUPPLEMENTARY METHODS

Robustness of Root Placement and Tree Topology to Lineage-specific Rate Heterogeneity

To assess the robustness of root placement to rate heterogeneity, both character-specific rate heterogeneity (CSRH) and lineage-specific rate heterogeneity (LSRH) was analyzed. Accounting for CSRH is described in the main text. The impact of LSRH on the placement of the root as well as the tree topology was assessed using relaxed-clock models. Relaxed-clock models account for LSRH as well as CSRH. Three different LSRH optimizations implemented in MrBayes 3.2, specifically, the CPP, TK02 and IGR models were analyzed under nonstationarity. The CPP model assumes that LSRH varies according to a compound Poisson process (Huelsenbeck, J.P., Larget, B., et al. 2000). Thorne–Kishino 2002 (TK02) model assumes a Brownian motion process in which LSRH is modeled according to an autocorrelated lognormal distribution (Thorne, J. and Kishino, H. 2002). The independent gamma rates (IGR) model (Lepage, T., Lawi, S., et al. 2006) assumes that each branch has an independent rate drawn from a gamma distribution. The difference between the models is different extent of rate variation allowed across the tree. For example the TK02 model assumes that LSRH between neighboring internodes of the tree are more similar than distant branches, where as the CPP models allows the rates to be change anywhere along the tree.

Robustness to Other Potential Errors

The robustness of the phylogenies inferred from the directional evolution-model against several potential systematic biases was assessed, and reported in independent studies (see below). Multiple datasets with varying taxon sampling as well as character sampling strategies were employed. In what follows, the term superkingdom will be used in place of

"domain of life" to represent the highest-ranking taxa (Archaea, Bacteria and Eukarya) in the global tree of life (ToL). This is to avoid potential confusion with the "domain of protein-structure".

(1) Robustness to species sampling. –Scalability of phylogenetic analysis as well as impact of species sampling on the directional model was compared by scale-up and scale down analyses. As for the former, phylogeny was estimated from four different datasets ranging from 75 species up to 336 species sampled across the tree of life (ToL). For the latter, i.e. scale-down analyses, phylogenies were estimated with as few as 30 species, sampled either across smaller clades (ranging from genera to phyla to kingdoms) or across the ToL (Harish, A., Tunlid, A., et al. 2013, Harish, A. and Kurland, C.G. 2017b).

In addition, here, further tests were conducted by excluding major taxa (e.g. all Eukarya) from the analyses to test if monophyly of the other taxa can still be recovered. Accordingly, trees were estimated for three combinations: (a) Archaea + Bacteria (Fig S2, A), (b) Archaea + Eukarya (Fig. S2, B) and (c) Bacteria + Eukarya (Fig. S2, C). Phylogenies were estimated in MrBayes using the relaxed-clock IGR model, since it is the best-fitting model (Fig. 6c), as described earlier. Here too Archaea, Bacteria and Eukarya were all monophyletic. The tree topologies for each of Archaea, Bacteria and Eukarya clades were almost identical compared to the full tree (Fig. 6a), except in one case. In the (Archaea + Eukarya) tree, the eukaryote clade was largely congruent.

(2) Robustness to character sampling. –This was assessed by the inclusion or exclusion of Superkingdom-specific characters (i.e. protein-domains), based on the broad phyletic distribution patterns. The total number of unique protein-domains varies extensively between Archaea, Bacteria and Eukarya, such that the least number of domains are shared amongst the species of archaea, followed by bacteria and the most number of domains shared between species of eukaryotes. Therefore, Superkingdom-specific protein-domains were

excluded from the analysis to assess potential bias in the root placement due to the biased distributions (Harish, A., Tunlid, A., et al. 2013, Harish, A. and Kurland, C.G. 2017a).

(3) Robustness to small (or large) genome-size bias. —The genomic composition of protein-domains is a reasonable proxy for the effective genome size measured as the number of proteins in a genome (or species) (Harish, A. and Kurland, C.G. 2017b). Accordingly the number of distinct domains in the each species sampled from archaea and bacteria is smaller, on average, compared to that of eukaryotes species. Therefore, robustness against potential artifacts arising solely due to genome size bias, e.g. 'small genome attraction' or 'large genome attraction' artifacts was assessed (Harish, A., Tunlid, A., et al. 2013, Harish, A. and Kurland, C.G. 2017a).

(4) Uncertainty in presence—absence coding (ascertainment bias). —Artifacts in SCOP-domain identification using hidden Markov models (HMMs) can affect the number of characters (domains) that can be scored for each species. For example false positives can inflate the number of 'presence' states while false negatives can inflate the number 'absence' states. Such character 'coding bias' can arise either due to suboptimal HMMs or due to a higher than expected sequence divergence in certain species or both. False positive identifications due to suboptimal HMMs are insignificant (~2% of all HMMs in SUPERFAMILY HMM library) (Gough, J., Karplus, K., et al. 2001, Pethica, R.B., Levitt, M., et al. 2012). However, estimating false negatives is nontrivial. Therefore false negatives were simulated by re-coding 'presence' states (1s) as 'absence' states (0s). Root placement as well as tree topology was robust to re-coding a large fraction (between 30% and 60%) (Harish, A. and Kurland, C.G. 2017c).

(5) Quality of genome sequence data/annotations. —The quality and consistency of genome annotation in terms of individual protein sequences and completeness of protein cohorts in a genome can often vary (Zaucha, J., Stahlhacke, J., et al. 2015) and can relate to

all of the biases explained above. Extensive analysis of multiple random samplings showed that the variation affects estimates of the domain abundance (copy number variation) but not of domain occurrence (presence-absence) analyses (Harish, A., Tunlid, A., et al. 2013, Harish, A. and Kurland, C.G. 2017b).

Altogether, the robustness of both root placement as well as the tree topology of the global ToL inferred from the SCOP-domain datasets against several potential phylogenetic artifacts were assessed. These comprehensive tests account for both stochastic errors as well as systematic biases.

Supplementary References

Gough J, Karplus K, Hughey R, Chothia C. 2001. Assignment of homology to genome sequences using a library of hidden Markov models that represent all proteins of known structure. Journal of Molecular Biology, 313:903-919.

Harish A, Kurland CG. 2017a. Akaryotes and Eukaryotes are independent descendants of a universal common ancestor. Biochimie, 138:168-183.

Harish A, Kurland CG. 2017b. Empirical genome evolution models root the tree of life. Biochimie, 138:137-155.

Harish A, Kurland CG. 2017c. Mitochondria are not captive bacteria. Journal of Theoretical Biology, 434:88-98.

Harish A, Tunlid A, Kurland CG. 2013. Rooted phylogeny of the three superkingdoms. Biochimie, 95:1593-1604.

Huelsenbeck JP, Larget B, Swofford D. 2000. A compound Poisson process for relaxing the molecular clock. Genetics, 154:1879-1892.

Lepage T, Lawi S, Tupper P, Bryant D. 2006. Continuous and tractable models for the variation of evolutionary rates. Mathematical biosciences, 199:216-233.

Pethica RB, Levitt M, Gough J. 2012. Evolutionarily consistent families in SCOP: Sequence, structure and function. BMC Structural Biology, 12.

Thorne J, Kishino H. 2002. Divergence time estimation and rate evolution with multilocus data sets. Syst. Biol, 51:689-702.

Zaucha J, Stahlhacke J, Oates ME, Thurlby N, Rackham OJ, Fang H, Smithers B, Gough J. 2015. A proteome quality index. Environmental microbiology, 17:4-9.

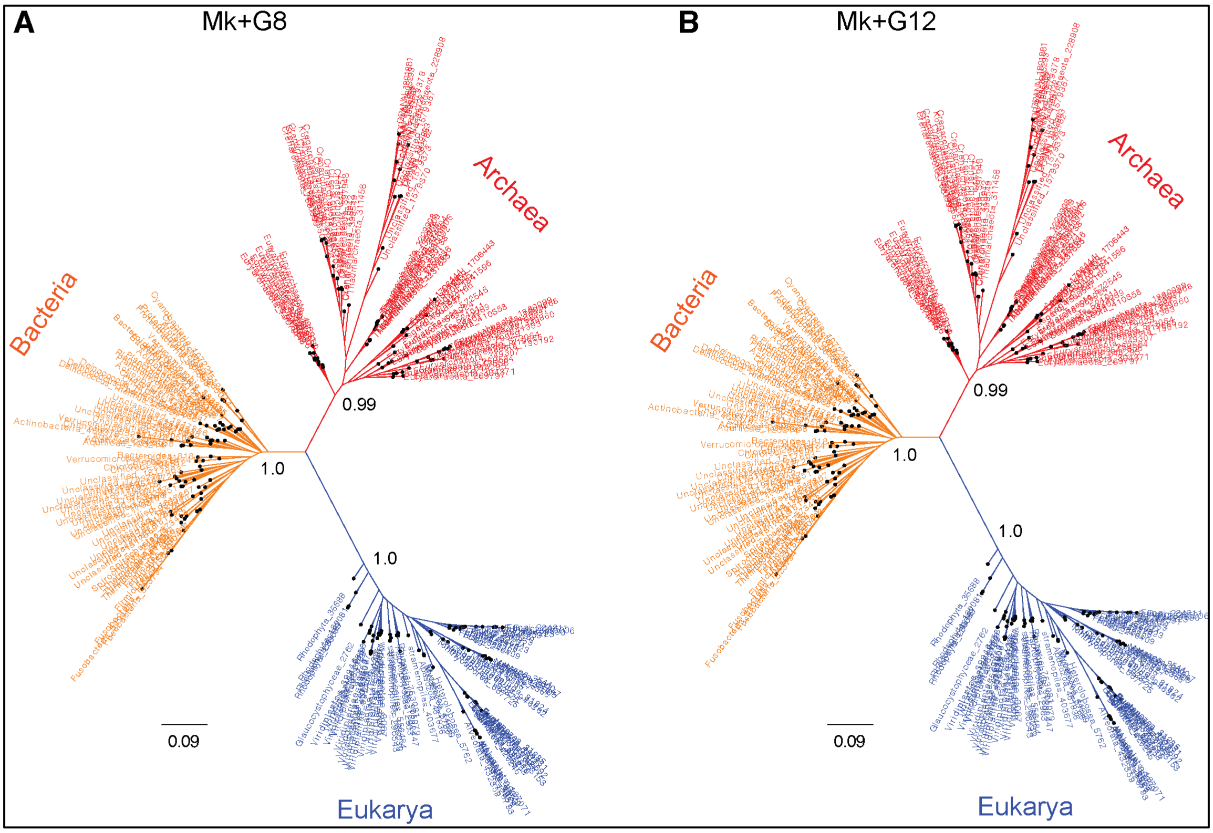

SI Fig. 1 Unrooted genome trees derived from rate-heterogeneous versions of the Mk model. (A) Unrooted tree estimated from Mk+G8 model and (B) from Mk+G12 model. Scale bars represent expected number of changes per character. Branch support (posterior probability) is shown only for the major branches.

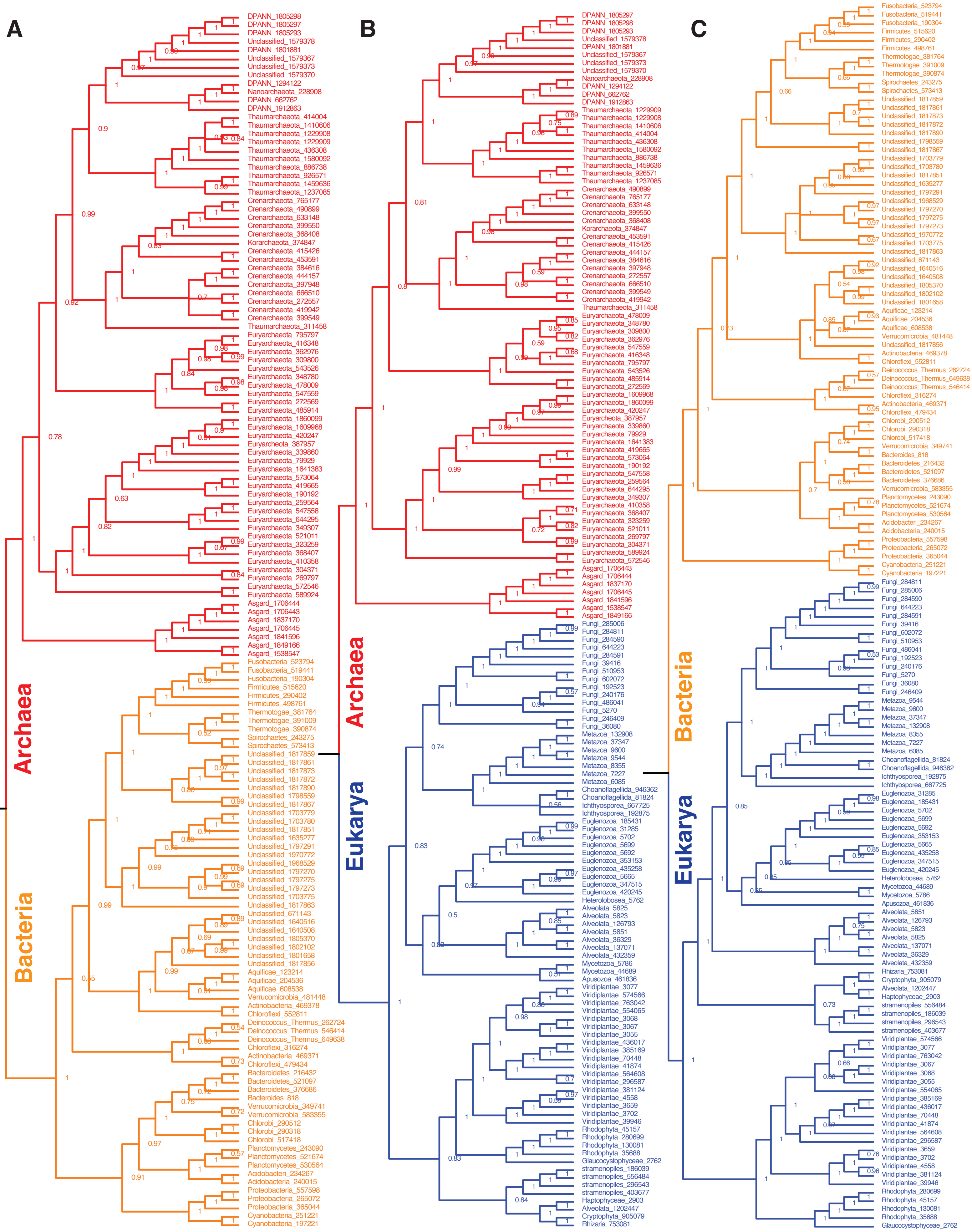
A
B
C
Archaea
Bacteria
Archaea
Eukarya
Bacteria
Eukarya

Figure S2 Impact of excluding major taxa on rooting or monophyly. Phylogenies were estimated after deleting all the eukaryotes, bacteria and archaea. Accordingly, trees were estimated for three combinations: (A) Archaea + Bacteria (B) Archaea + Eukarya and (C) Bacteria + Eukarya. Phylogenies were estimated in MrBayes using the relaxed-clock IGR model.

Table S1. List of species sampled in the SCOP-II dataset

Tree label	Taxonomy ID	Scientific name	Taxonomic group	Superkingdom
Asgard_1538547	1538547	*Lokiarchaeum sp. GC14_75*	Asgard	Archaea
Asgard_1706443	1706443	*Candidatus Thorarchaeota archaeon SMTZ-45*	Asgard	Archaea
Asgard_1706444	1706444	*Candidatus Thorarchaeota archaeon SMTZ1-45*	Asgard	Archaea
Asgard_1706445	1706445	*Candidatus Thorarchaeota archaeon SMTZ1-83*	Asgard	Archaea
Asgard_1837170	1837170	*Candidatus Thorarchaeota archaeon AB_25*	Asgard	Archaea
Asgard_1841596	1841596	*Candidatus Heimdallarchaeota archaeon AB_125*	Asgard	Archaea
Asgard_1849166	1849166	*Candidatus Lokiarchaeota archaeon CR_4*	Asgard	Archaea
Crenarchaeota_272557	272557	*Aeropyrum pernix*	Crenarchaeota	Archaea
Crenarchaeota_368408	368408	*Thermofilum pendens*	Crenarchaeota	Archaea
Crenarchaeota_384616	384616	*Pyrobaculum islandicum*	Crenarchaeota	Archaea
Crenarchaeota_397948	397948	*Caldivirga maquilingensis*	Crenarchaeota	Archaea
Crenarchaeota_399549	399549	*Metallosphaera sedula*	Crenarchaeota	Archaea
Crenarchaeota_399550	399550	*Staphylothermus marinus*	Crenarchaeota	Archaea
Crenarchaeota_415426	415426	*Hyperthermus butylicus*	Crenarchaeota	Archaea
Crenarchaeota_419942	419942	*Sulfolobus islandicus*	Crenarchaeota	Archaea
Crenarchaeota_444157	444157	*Pyrobaculum neutrophilum*	Crenarchaeota	Archaea
Crenarchaeota_453591	453591	*Ignicoccus hospitalis*	Crenarchaeota	Archaea
Crenarchaeota_490899	490899	*Desulfurococcus amylolyticus*	Crenarchaeota	Archaea
Crenarchaeota_633148	633148	*Thermosphaera aggregans*	Crenarchaeota	Archaea
Crenarchaeota_666510	666510	*Acidilobus saccharovorans*	Crenarchaeota	Archaea
Crenarchaeota_765177	765177	*Desulfurococcus mucosus*	Crenarchaeota	Archaea
DPANN_662762	662762	*Candidatus Parvarchaeum acidophilus ARMAN-5*	DPANN	Archaea
DPANN_1294122	1294122	*Candidatus Nanobsidianus stetteri*	DPANN	Archaea
DPANN_1801881	1801881	*Candidatus Pacearchaeota archaeon RBG_13_36_9*	DPANN	Archaea
DPANN_1805293	1805293	*Candidatus Pacearchaeota archaeon CG1_02_30_18*	DPANN	Archaea
DPANN_1805297	1805297	*Candidatus Pacearchaeota archaeon CG1_02_35_32*	DPANN	Archaea
DPANN_1805298	1805298	*Candidatus Pacearchaeota archaeon CG1_02_39_14*	DPANN	Archaea
DPANN_1912863	1912863	*Candidatus Micrarchaeum acidiphilum ARMAN-1*	DPANN	Archaea
Euryarchaeota_79929	79929	*Methanothermobacter marburgensis*	Euryarchaeota	Archaea
Euryarchaeota_190192	190192	*Methanopyrus kandleri*	Euryarchaeota	Archaea
Euryarchaeota_259564	259564	*Methanococcoides burtonii*	Euryarchaeota	Archaea
Euryarchaeota_269797	269797	*Methanosarcina barkeri*	Euryarchaeota	Archaea
Euryarchaeota_272569	272569	*Haloarcula marismortui*	Euryarchaeota	Archaea
Euryarchaeota_304371	304371	*Methanocella paludicola*	Euryarchaeota	Archaea
Euryarchaeota_309800	309800	*Haloferax volcanii*	Euryarchaeota	Archaea
Euryarchaeota_323259	323259	*Methanospirillum hungatei JF-1*	Euryarchaeota	Archaea
Euryarchaeota_339860	339860	*Methanosphaera stadtmanae*	Euryarchaeota	Archaea
Euryarchaeota_348780	348780	*Natronomonas pharaonis*	Euryarchaeota	Archaea
Euryarchaeota_349307	349307	*Methanosaeta thermophila*	Euryarchaeota	Archaea
Euryarchaeota_362976	362976	*Haloquadratum walsbyi*	Euryarchaeota	Archaea
Euryarchaeota_368407	368407	*Methanoculleus marisnigri*	Euryarchaeota	Archaea
Euryarchaeota_410358	410358	*Methanocorpusculum labreanum*	Euryarchaeota	Archaea
Euryarchaeota_416348	416348	*Halorubrum lacusprofundi*	Euryarchaeota	Archaea
Euryarchaeota_419665	419665	*Methanococcus aeolicus*	Euryarchaeota	Archaea
Euryarchaeota_420247	420247	*Methanobrevibacter smithii*	Euryarchaeota	Archaea
Euryarchaeota_478009	478009	*Halobacterium salinarum*	Euryarchaeota	Archaea
Euryarchaeota_485914	485914	*Halomicrobium mukohataei*	Euryarchaeota	Archaea
Euryarchaeota_521011	521011	*Methanosphaerula palustris*	Euryarchaeota	Archaea
Euryarchaeota_543526	543526	*Haloterrigena turkmenica*	Euryarchaeota	Archaea
Euryarchaeota_547558	547558	*Methanohalophilus mahii*	Euryarchaeota	Archaea
Euryarchaeota_547559	547559	*Natrialba magadii*	Euryarchaeota	Archaea
Euryarchaeota_572546	572546	*Archaeoglobus profundus*	Euryarchaeota	Archaea
Euryarchaeota_573064	573064	*Methanocaldococcus fervens*	Euryarchaeota	Archaea
Euryarchaeota_589924	589924	*Ferroglobus placidus*	Euryarchaeota	Archaea
Euryarchaeota_644295	644295	*Methanohalobium evestigatum*	Euryarchaeota	Archaea
Euryarchaeota_795797	795797	*Halalkalicoccus jeotgali*	Euryarchaeota	Archaea
Euryarchaeota_1609968	1609968	*Methanobrevibacter sp. YE315*	Euryarchaeota	Archaea
Euryarchaeota_1641383	1641383	*Methanobacterium sp. 42_16*	Euryarchaeota	Archaea
Euryarchaeota_1860099	1860099	*Methanobrevibacter sp. A27*	Euryarchaeota	Archaea
Euryarcheota_387957	387957	*Methanobrevibacter sp. 87.7*	Euryarcheota	Archaea
Korarchaeota_374847	374847	*Korarchaeum cryptofilum*	Korarchaeota	Archaea
Nanoarchaeota_228908	228908	*Nanoarchaeum equitans*	Nanoarchaeota	Archaea
Thaumarchaeota_311458	311458	*Candidatus Caldiarchaeum subterraneum*	Thaumarchaeota	Archaea
Thaumarchaeota_414004	414004	*Cenarchaeum symbiosum*	Thaumarchaeota	Archaea
Thaumarchaeota_436308	436308	*Nitrosopumilus maritimus*	Thaumarchaeota	Archaea
Thaumarchaeota_886738	886738	*Candidatus Nitrosoarchaeum limnia SFB1*	Thaumarchaeota	Archaea
Thaumarchaeota_926571	926571	*Nitrososphaera viennensis EN76*	Thaumarchaeota	Archaea
Thaumarchaeota_1229908	1229908	*Candidatus Nitrosopumilus koreensis AR1*	Thaumarchaeota	Archaea
Thaumarchaeota_1229909	1229909	*Candidatus Nitrosopumilus sediminis*	Thaumarchaeota	Archaea
Thaumarchaeota_1237085	1237085	*Nitrososphaera gargensis*	Thaumarchaeota	Archaea
Thaumarchaeota_1410606	1410606	*Candidatus Nitrosopelagicus brevis*	Thaumarchaeota	Archaea
Thaumarchaeota_1459636	1459636	*Candidatus Nitrososphaera evergladensis SR1*	Thaumarchaeota	Archaea
Thaumarchaeota_1580092	1580092	*Candidatus Nitrosopumilus adriaticus*	Thaumarchaeota	Archaea
Unclassified_1579367	1579367	*archaeon GW2011_AR5*	Unclassified	Archaea

Unclassified_1579370	1579370	*archaeon GW2011_AR10*	Unclassified	Archaea
Unclassified_1579373	1579373	*archaeon GW2011_AR15*	Unclassified	Archaea
Unclassified_1579378	1579378	*archaeon GW2011_AR20*	Unclassified	Archaea
Acidobacteri_234267	234267	*Solibacter usitatus*	Acidobacteri	Bacteria
Acidobacteria_240015	240015	*Acidobacterium capsulatum*	Acidobacteria	Bacteria
Actinobacteria_469371	469371	*Thermobispora bispora*	Actinobacteria	Bacteria
Actinobacteria_469378	469378	*Cryptobacterium curtum*	Actinobacteria	Bacteria
Aquificae_123214	123214	*Persephonella marina*	Aquificae	Bacteria
Aquificae_204536	204536	*Sulfurihydrogenibium azorense*	Aquificae	Bacteria
Aquificae_608538	608538	*Hydrogenobacter thermophilus*	Aquificae	Bacteria
Bacteroides_818	818	*Bacteroides thetaiotaomicron*	Bacteroides	Bacteria
Bacteroidetes_216432	216432	*Croceibacter atlanticus*	Bacteroidetes	Bacteria
Bacteroidetes_376686	376686	*Flavobacterium johnsoniae*	Bacteroidetes	Bacteria
Bacteroidetes_521097	521097	*Capnocytophaga ochracea*	Bacteroidetes	Bacteria
Chlorobi_290318	290318	*Chlorobium phaeovibrioides*	Chlorobi	Bacteria
Chlorobi_290512	290512	*Prosthecochloris aestuarii*	Chlorobi	Bacteria
Chlorobi_517418	517418	*Chloroherpeton thalassium*	Chlorobi	Bacteria
Chloroflexi_316274	316274	*Herpetosiphon aurantiacus*	Chloroflexi	Bacteria
Chloroflexi_479434	479434	*Sphaerobacter thermophilus*	Chloroflexi	Bacteria
Chloroflexi_552811	552811	*Dehalogenimonas lykanthroporepellens*	Chloroflexi	Bacteria
Cyanobacteria_197221	197221	*Thermosynechococcus elongatus*	Cyanobacteria	Bacteria
Cyanobacteria_251221	251221	*Gloeobacter violaceus*	Cyanobacteria	Bacteria
Deinococcus_Thermus_262724	262724	*Thermus thermophilus*	Deinococcus_Thermus	Bacteria
Deinococcus_Thermus_546414	546414	*Deinococcus deserti*	Deinococcus_Thermus	Bacteria
Deinococcus_Thermus_649638	649638	*Truepera radiovictrix*	Deinococcus_Thermus	Bacteria
Firmicutes_290402	290402	*Clostridium beijerinckii*	Firmicutes	Bacteria
Firmicutes_498761	498761	*Heliobacterium modesticaldum*	Firmicutes	Bacteria
Firmicutes_515620	515620	*Eubacterium eligens*	Firmicutes	Bacteria
Fusobacteria_190304	190304	*Fusobacterium nucleatum subsp. nucleatum*	Fusobacteria	Bacteria
Fusobacteria_519441	519441	*Streptobacillus moniliformis*	Fusobacteria	Bacteria
Fusobacteria_523794	523794	*Leptotrichia buccalis*	Fusobacteria	Bacteria
Planctomycetes_243090	243090	*Rhodopirellula baltica*	Planctomycetes	Bacteria
Planctomycetes_521674	521674	*Planctopirus limnophila*	Planctomycetes	Bacteria
Planctomycetes_530564	530564	*Pirellula staleyi*	Planctomycetes	Bacteria
Proteobacteria_265072	265072	*Methylobacillus flagellatus*	Proteobacteria	Bacteria
Proteobacteria_365044	365044	*Polaromonas naphthalenivorans*	Proteobacteria	Bacteria
Proteobacteria_557598	557598	*Laribacter hongkongensis*	Proteobacteria	Bacteria
Spirochaetes_243275	243275	*Treponema denticola*	Spirochaetes	Bacteria
Spirochaetes_573413	573413	*Sediminispirochaeta smaragdinae*	Spirochaetes	Bacteria
Thermotogae_381764	381764	*Fervidobacterium nodosum*	Thermotogae	Bacteria
Thermotogae_390874	390874	*Thermotoga petrophila*	Thermotogae	Bacteria
Thermotogae_391009	391009	*Thermosipho melanesiensis*	Thermotogae	Bacteria
Unclassified_671143	671143	*Candidatus Methylomirabilis oxyfera*	Unclassified	Bacteria
Unclassified_1635277	1635277	*candidate division TA06 bacterium 34_109*	Unclassified	Bacteria
Unclassified_1640508	1640508	*Candidatus Dadabacteria bacterium CSP1-2*	Unclassified	Bacteria
Unclassified_1640516	1640516	*candidate division NC10 bacterium CSP1-5*	Unclassified	Bacteria
Unclassified_1703775	1703775	*candidate division WOR_1 bacterium DG_54_3*	Unclassified	Bacteria
Unclassified_1703779	1703779	*candidate division WOR_3 bacterium SM23_42*	Unclassified	Bacteria
Unclassified_1703780	1703780	*candidate division WOR_3 bacterium SM23_60*	Unclassified	Bacteria
Unclassified_1797270	1797270	*Candidatus Aminicenantes bacterium RBG_13_63_10*	Unclassified	Bacteria
Unclassified_1797273	1797273	*Candidatus Aminicenantes bacterium RBG_16_63_16*	Unclassified	Bacteria
Unclassified_1797275	1797275	*Candidatus Aminicenantes bacterium RBG_19FT_COMBO_58_17*	Unclassified	Bacteria
Unclassified_1797291	1797291	*Candidatus Atribacteria bacterium RBG_19FT_COMBO_35_14*	Unclassified	Bacteria
Unclassified_1798559	1798559	*candidate division KSB1 bacterium RBG_16_48_16*	Unclassified	Bacteria
Unclassified_1801658	1801658	*candidate division NC10 bacterium RIFCSPLOWO2_02_FULL_66_22*	Unclassified	Bacteria
Unclassified_1802102	1802102	*Candidatus Rokubacteria bacterium RIFCSPHIGHO2_02_FULL_73_26*	Unclassified	Bacteria
Unclassified_1805370	1805370	*Candidatus Rokubacteria bacterium 13_2_20CM_2_70_11*	Unclassified	Bacteria
Unclassified_1817851	1817851	*Candidatus Edwardsbacteria bacterium GWF2_54_11*	Unclassified	Bacteria
Unclassified_1817856	1817856	*Candidatus Eisenbacteria bacterium RBG_16_71_46*	Unclassified	Bacteria
Unclassified_1817859	1817859	*Candidatus Firestonebacteria bacterium RIFOXYA2_FULL_40_8*	Unclassified	Bacteria
Unclassified_1817861	1817861	*Candidatus Firestonebacteria bacterium RIFOXYC2_FULL_39_67*	Unclassified	Bacteria
Unclassified_1817863	1817863	*Candidatus Fischerbacteria bacterium RBG_13_37_8*	Unclassified	Bacteria
Unclassified_1817867	1817867	*Candidatus Glassbacteria bacterium RIFCSPLOWO2_12_FULL_58_11*	Unclassified	Bacteria
Unclassified_1817872	1817872	*Candidatus Margulisbacteria bacterium GWE2_39_32*	Unclassified	Bacteria
Unclassified_1817873	1817873	*Candidatus Margulisbacteria bacterium GWF2_35_9*	Unclassified	Bacteria
Unclassified_1817890	1817890	*Candidatus Raymondbacteria bacterium RIFOXYD12_FULL_49_13*	Unclassified	Bacteria
Unclassified_1968529	1968529	*Candidatus Aminicenantes bacterium 4484_214*	Unclassified	Bacteria
Unclassified_1970772	1970772	*candidate division KSB1 bacterium 4484_87*	Unclassified	Bacteria
Verrucomicrobia_349741	349741	*Akkermansia muciniphila*	Verrucomicrobia	Bacteria
Verrucomicrobia_481448	481448	*Methylacidiphilum infernorum*	Verrucomicrobia	Bacteria
Verrucomicrobia_583355	583355	*Coraliomargarita akajimensis*	Verrucomicrobia	Bacteria
Alveolata_5823	5823	*Plasmodium berghei*	Alveolata	Eukarya
Alveolata_5825	5825	*Plasmodium chabaudi*	Alveolata	Eukarya
Alveolata_5851	5851	*Plasmodium knowlesi*	Alveolata	Eukarya
Alveolata_36329	36329	*Plasmodium falciparum*	Alveolata	Eukarya

Alveolata_126793	126793	*Plasmodium vivax*	Alveolata	Eukarya
Alveolata_137071	137071	*Plasmodium falciparum*	Alveolata	Eukarya
Alveolata_432359	432359	*Toxoplasma gondii*	Alveolata	Eukarya
Alveolata_1202447	1202447	*Symbiodinium minutum*	Alveolata	Eukarya
Choanoflagellida_81824	81824	*Monosiga brevicollis*	Choanoflagellida	Eukarya
Choanoflagellida_946362	946362	*Salpingoeca rosetta*	Choanoflagellida	Eukarya
Cryptophyta_905079	905079	*Guillardia theta CCMP2712*	Cryptophyta	Eukarya
Euglenozoa_5665	5665	*Leishmania mexicana*	Euglenozoa	Eukarya
Euglenozoa_5692	5692	*Trypanosoma congolense*	Euglenozoa	Eukarya
Euglenozoa_5699	5699	*Trypanosoma vivax*	Euglenozoa	Eukarya
Euglenozoa_5702	5702	*Trypanosoma brucei brucei*	Euglenozoa	Eukarya
Euglenozoa_31285	31285	*Trypanosoma brucei gambiense*	Euglenozoa	Eukarya
Euglenozoa_185431	185431	*Trypanosoma brucei brucei*	Euglenozoa	Eukarya
Euglenozoa_347515	347515	*Leishmania major strain Friedlin*	Euglenozoa	Eukarya
Euglenozoa_353153	353153	*Trypanosoma cruzi*	Euglenozoa	Eukarya
Euglenozoa_420245	420245	*Leishmania braziliensis*	Euglenozoa	Eukarya
Euglenozoa_435258	435258	*Leishmania infantum*	Euglenozoa	Eukarya
Fungi_5270	5270	*Ustilago maydis*	Fungi	Eukarya
Fungi_36080	36080	*Mucor circinelloides*	Fungi	Eukarya
Fungi_39416	39416	*Tuber melanosporum*	Fungi	Eukarya
Fungi_192523	192523	*Agaricus bisporus var. bisporus*	Fungi	Eukarya
Fungi_240176	240176	*Coprinopsis cinerea*	Fungi	Eukarya
Fungi_246409	246409	*Rhizopus delemar*	Fungi	Eukarya
Fungi_284590	284590	*Kluyveromyces lactis*	Fungi	Eukarya
Fungi_284591	284591	*Yarrowia lipolytica*	Fungi	Eukarya
Fungi_284811	284811	*Ashbya gossypii*	Fungi	Eukarya
Fungi_285006	285006	*Saccharomyces cerevisiae*	Fungi	Eukarya
Fungi_486041	486041	*Laccaria bicolor*	Fungi	Eukarya
Fungi_510953	510953	*Neurospora discreta*	Fungi	Eukarya
Fungi_602072	602072	*Aspergillus carbonarius*	Fungi	Eukarya
Fungi_644223	644223	*Komagataella phaffii*	Fungi	Eukarya
Glaucocystophyceae_2762	2762	*Cyanophora paradoxa*	Glaucocystophyceae	Eukarya
Haptophyceae_2903	2903	*Emiliania huxleyi*	Haptophyceae	Eukarya
Ichthyosporea_192875	192875	*Capsaspora owczarzaki*	Ichthyosporea	Eukarya
Ichthyosporea_667725	667725	*Sphaeroforma arctica JP610*	Ichthyosporea	Eukarya
Metazoa_6085	6085	*Hydra vulgaris*	Metazoa	Eukarya
Metazoa_7227	7227	*Drosophila melanogaster*	Metazoa	Eukarya
Metazoa_8355	8355	*Xenopus laevis*	Metazoa	Eukarya
Metazoa_9544	9544	*Macaca mulatta*	Metazoa	Eukarya
Metazoa_9600	9600	*Pongo pygmaeus*	Metazoa	Eukarya
Metazoa_37347	37347	*Tupaia belangeri*	Metazoa	Eukarya
Metazoa_132908	132908	*Pteropus vampyrus*	Metazoa	Eukarya
Rhizaria_753081	753081	*Bigelowiella natans*	Rhizaria	Eukarya
Rhodophyta_35688	35688	*Porphyridium purpureum*	Rhodophyta	Eukarya
Rhodophyta_45157	45157	*Cyanidioschyzon merolae*	Rhodophyta	Eukarya
Rhodophyta_130081	130081	*Galdieria sulphuraria*	Rhodophyta	Eukarya
Rhodophyta_280699	280699	*Cyanidioschyzon merolae*	Rhodophyta	Eukarya
stramenopiles_186039	186039	*Fragilariopsis cylindrus*	stramenopiles	Eukarya
stramenopiles_296543	296543	*Thalassiosira pseudonana CCMP1335*	stramenopiles	Eukarya
stramenopiles_403677	403677	*Phytophthora infestans*	stramenopiles	Eukarya
stramenopiles_556484	556484	*Phaeodactylum tricornutum*	stramenopiles	Eukarya
Viridiplantae_3055	3055	*Chlamydomonas reinhardtii*	Viridiplantae	Eukarya
Viridiplantae_3067	3067	*Volvox carteri*	Viridiplantae	Eukarya
Viridiplantae_3068	3068	*Volvox carteri f. nagariensis*	Viridiplantae	Eukarya
Viridiplantae_3077	3077	*Chlorella vulgaris*	Viridiplantae	Eukarya
Viridiplantae_3659	3659	*Cucumis sativus*	Viridiplantae	Eukarya
Viridiplantae_3702	3702	*Arabidopsis thaliana*	Viridiplantae	Eukarya
Viridiplantae_4558	4558	*Sorghum bicolor*	Viridiplantae	Eukarya
Viridiplantae_39946	39946	*Oryza sativa subsp. indica*	Viridiplantae	Eukarya
Viridiplantae_41874	41874	*Bathycoccus prasinos*	Viridiplantae	Eukarya
Viridiplantae_70448	70448	*Ostreococcus tauri*	Viridiplantae	Eukarya
Viridiplantae_296587	296587	*Micromonas commoda*	Viridiplantae	Eukarya
Viridiplantae_381124	381124	*Zea mays subsp. mays*	Viridiplantae	Eukarya
Viridiplantae_385169	385169	*Ostreococcus sp*	Viridiplantae	Eukarya
Viridiplantae_436017	436017	*Ostreococcus lucimarinus*	Viridiplantae	Eukarya
Viridiplantae_554065	554065	*Chlorella variabilis*	Viridiplantae	Eukarya
Viridiplantae_564608	564608	*Micromonas pusilla*	Viridiplantae	Eukarya
Viridiplantae_574566	574566	*Coccomyxa subellipsoidea*	Viridiplantae	Eukarya
Viridiplantae_763042	763042	*Asterochloris sp*	Viridiplantae	Eukarya

Table S2. List of sequence evolution models evaluated for the core-genes-I dataset

Base Model	Heterogeneity	K	Likelihood	LLR	AIC	BIC
LG	+G+I	87	-492883	0	985940	986553
LG	+G+I+F	106	-493145	262	986502	987250
LG	+G	86	-493206	324	986585	987191
LG	+G+F	105	-493420	538	987051	987791
RtREV	+G+I+F	106	-494212	1330	988637	989385
WAG	+G+I+F	106	-496011	3128	992234	992982
WAG	+G+I	87	-496884	4001	993942	994555
VT	+G+I+F	106	-496978	4096	994169	994916
Blosum62	+G+I	87	-497004	4121	994182	994796
VT	+G+I	87	-497243	4360	994660	995274
RtREV	+G+I	87	-497421	4538	995016	995630
Blosum62	+G+I+F	106	-497536	4654	995285	996033
CpREV	+G+I	87	-498666	5783	997506	998120
CpREV	+G+I+F	106	-498692	5809	997596	998344
JTT	+G+I	87	-501937	9055	1004049	1004662
JTT	+G+I+F	106	-501986	9103	1004183	1004931
MtZoa	+G+I+F	106	-502729	9846	1005670	1006418
DCMut	+G+I+F	106	-503422	10539	1007055	1007803
Dayhoff	+G+I+F	106	-503425	10543	1007063	1007811
MtREV	+G+I+F	106	-505701	12818	1011614	1012362
MtArt	+G+I+F	106	-507424	14541	1015060	1015807
Flu	+G+I+F	106	-510433	17550	1021078	1021826
HIVb	+G+I+F	106	-514167	21284	1028546	1029294
HIVb	+G+I	87	-514445	21563	1029065	1029679
AB	+G+I+F	106	-515188	22305	1030588	1031336
MtMam	+G+I+F	106	-520429	27546	1041069	1041817
HIVw	+G+I+F	106	-532211	39328	1064634	1065381

Base Model = Generalized empirical amino acid exchange rate (probability)

Heterogeneity = Parameter for approximating site-specific variation

+GX; where G is discrete Gamma model, X is no. of categories (default X=4)

+I; proportion of invariant sites

+F; empirical amino acid frequencies estimated from the alignment

K = Number of parameters

Likelihood = Raw likelihood score

LLR = Log likelihood ratio given as the difference from the best fitting model

AIC = Akaike information criterion

BIC = Bayesian information criterion

Table S3. List of sequence evolution models evaluated for the core-genes-II dataset

**Base Model**	**Heterogeneity**	**K**	**Likelihood**	**LLR**	**AIC**	**BIC**
LG	+G+I	191	-1136837	0	2274056	2275431
LG	+G+I+F	210	-1137034	197	2274488	2276000
LG	+G	190	-1137480	643	2275339	2276707
LG	+G+F	209	-1137609	773	2275637	2277141
RtREV	+G+I+F	210	-1139815	2978	2280050	2281562
WAG	+G+I+F	210	-1143506	6669	2287432	2288944
VT	+G+I+F	210	-1145801	8964	2292021	2293533
WAG	+G+I	191	-1146408	9571	2293198	2294572
Blosum62	+G+I	191	-1146621	9784	2293624	2294999
VT	+G+I	191	-1146865	10028	2294112	2295487
Blosum62	+G+I+F	210	-1147798	10961	2296015	2297527
CpREV	+G+I	191	-1149437	12600	2299256	2300630
CpREV	+G+I+F	210	-1151328	14491	2303077	2304588
JTT	+G+I+F	210	-1156779	19942	2313978	2315490
JTT	+G+I	191	-1157879	21043	2316141	2317516
DCMut	+G+I+F	210	-1158756	21919	2317931	2319443
Dayhoff	+G+I+F	210	-1158763	21926	2317947	2319458
MtZoa	+G+I+F	210	-1159644	22807	2319708	2321220
MtREV	+G+I+F	210	-1167250	30413	2334921	2336432
MtArt	+G+I+F	210	-1170882	34046	2342185	2343696
Flu	+G+I+F	210	-1177036	40199	2354492	2356004
HIVb	+G+I+F	210	-1186361	49524	2373143	2374654
AB	+G+I+F	210	-1188156	51319	2376731	2378242
MtMam	+G+I+F	210	-1202305	65468	2405029	2406541
HIVw	+G+I+F	210	-1229597	92760	2459614	2461126

Base Model = Generalized empirical amino acid exchange rate (probability)

Heterogeneity = Parameter for approximating site-specific variation

+GX; where G is discrete Gamma model, X is no. of categories (default X=4)

+I; proportion of invariant sites

+F; empirical amino acid frequencies estimated from the alignment

K = Number of parameters

Likelihood = Raw likelihood score

LLR = Log likelihood ratio given as the difference from the best fitting model

AIC = Akaike information criterion

BIC = Bayesian information criterion
